# Some Aspects of Intestinal Obstruction

**Published:** 1908-07-04

**Authors:** 


### SOME ASPECTS OF INTESTINAL OBSTRUCTION.

### CARCINOMA AS IT AFFECTS THE DIFFERENT REGIONS OF THE BOWEL.

In carcinoma of the large intestine spontaneous pain, which is constantly referred to the same region of the abdomen, is a prominent symptom. It is usually increased by pressure over the painful area, whether a tumour can be palpated or not, and is most often due to circumscribed peritonitis in the neighbourhood of the tumour. But it must be remembered that the situation of this pain is of no value in localising the site of the tumour. As Treves has pointed out, the localisation of pain by a patient is a matter of education; by an unconscious process of repeated comparison, the individual acquires a knowledge of his own skin, so far as it is concerned as a vehicle for sensation. He has, however, much fewer experiences of intestinal pain, and he is unable to associate such pain with any particular region, because he cannot corroborate his deductions by eyesight. The symptom is of little value, therefore, in localising the stenosis; but, for what it is worth, it is obviously of more value in growths of the large intestine, which are fixed and constant in position, than it is in similar conditions of the small intestine.

The presence of blood and pus in the stools is an indication that the surface of the growth is bleeding and ulcerated, owing to the passage of hard fæcal masses over the stenosed area. The condition is almost pathognomonic of large intestine stenosis, since the only other conditions which cause a discharge of blood and purulent matter are colitis and dysenteric ulcer, and these can easily be excluded. If disintegration of the growth is proceeding the odour of the dejecta is characteristic of gangrenous material, and if small particles of growth can be microscopically demonstrated in the stools, the diagnosis of carcinoma of the large intestine is clinched.

The presence of a palpable tumour in a case where carcinoma is suspected materially strengthens the diagnosis. Of course, carcinoma may be present although no tumour can be felt, but in the majority of cases careful examination will reveal the existence of a swelling, especially if, as is often the case, the abdominal parietes are thin. The tumour may vary in size from that of a walnut to that of a fœtal head. It usually feels solid and hard like cartilage, and is moderately tender on pressure. It is often astonishingly mobile, especially if it is situated in the transverse colon or sigmoid. It is subject to great variations in shape and size on account of the large amount of fæcal matter which collects above it; it is, therefore, quite impossible to estimate the size of the actual growth from the size of the tumour felt. Many striking errors of diagnosis have been made in this way, and tumours which were composed entirely of fæces have before now been operated upon under the impression that they were carcinomatous. In doubtful cases the differential diagnosis can often be cleared up by the administration of laxative purges and enemata, so long as the patient is kept under observation. The last reservation is important because it must be remembered that the administration of purges in a case which is really a carcinoma may lead to an attack of an acute obstruction. In some cases the differential diagnosis may be extraordinarily difficult, but the fundamental features of carcinoma of the large intestine are the pain, the presence of blood and pus in the fæces, and the existence of a palpable tumour in the course of the large intestine.

In carcinoma of the rectum the symptoms are essentially the same as those of carcinoma of the colon, but there is one great difference. If the rectum is digitally examined, the diagnosis of rectal carcinoma is very easy and very positive. In a case presenting symptoms of intestinal obstruction, a rectal examination should under no circumstances be omitted. Two special symptoms are more constantly associated with rectal cancer than with other intestinal carcinomata. They are hæmorrhoids, and a patulous sphincter with ballooning of the rectum. Every patient complaining of piles, especially if he be a man of middle age, should be rectally examined. The observance of this rule will save the surgeon from many errors of diagnosis. The piles are caused, of course, by obstruction to the return of blood along the hæmorrhoidal veins. The patulous sphincter and the ballooning of the rectum are the result of paralysis from interference with their nerve supply. On rectal examination the bowel is found to be empty and dilated to its utmost extent. The finger appears after passing the sphincter to come suddenly into an open space. The rectal wall is fixed in a stretched condition, its surface is smooth, and the normal rugæ have vanished.

Digital examination will give more or less complete information as to the size, outline, situation, and stage of development of the growth. The varieties of growth are the same as those found elsewhere in the intestine—an isolated nodule, a cauliflower excrescence, a hard, plate-like mass, or an annular stricture.

Duodenal carcinoma gives rise to symptoms of a somewhat anomalous nature. Pain is usually referred to the epigastric or right hypochondriac region. If a tumour can be felt, it will be in the right hypochondrium. It differs from the tumours formed by intestinal carcinoma elsewhere in being quite fixed, a fact which is caused by the manner in which the duodenum is bound down to the posterior abdominal wall by its peritoneal investment.

The symptoms are nearly all due to disturbance of the stomach functions, so that gastric disease is often simulated. The patient complains of loss of appetite, belching, vomiting, and in some cases of hæmatemesis. It is said that the exact site of the growth can be determined by the following symptoms. If it is situated above the ampulla of Vater, which guards the entrance of the common bile ducts, there is gastrectasis. Carcinoma actually involving the ampulla causes intense jaundice, while malignant disease below this causes bilious vomiting.

Carcinoma of the jejunum and ileum is rare. The only characteristic sign is the extraordinary mobility of the tumour formed by the growth, if it can be palpated at all. Carcinoma of the transverse colon is the only other form which is at all comparable to it in this respect.

